# Editorial: Regulating Liver Transcriptional Networks by Endocrine, Extracellular, and Intrinsic Cues

**DOI:** 10.3389/fendo.2019.00878

**Published:** 2019-12-13

**Authors:** Lars Grøntved, Ido Goldstein

**Affiliations:** ^1^Department of Biochemistry and Molecular Biology, Center for Functional Genomics and Tissue Plasticity, University of Southern Denmark, Odense, Denmark; ^2^Institute of Biochemistry, Food Science and Nutrition, The Robert H. Smith Faculty of Agriculture, Food and Environment, The Hebrew University of Jerusalem, Rehovot, Israel

**Keywords:** chromatin, liver, transcription, nuclear receptors, hormones

The many functions of the liver are regulated in response to extracellular cues (hormones, cytokines, metabolites etc.). The hepatic response to these signals is largely mediated by regulation of gene transcription that helps maintain homeostasis in a constantly changing environment. This is in addition to non-genomic effects of extracellular signals. This article collection deals with different aspects of hepatic gene regulation. Of particular interest to transcriptional regulation are nuclear receptors (NRs). NRs are a group of transcription factors (TFs) whose activity is controlled by steroid hormones and metabolites, leading to overt regulation of gene expression. Upon activation, NRs bind to their DNA sequence motif and initiate a set of events culminating in altered gene expression (1). NRs orchestrate gene transcription through interaction with a range of co-regulators who are themselves regulated by upstream signaling pathways sensing the metabolic status of the cells (2, 3). Thus, NRs and their co-regulators serve as a bridge between the extracellular milieu of signals and the cellular response to them.

An example for the effect NRs have on hepatic gene expression is given in the study by Murani et al. where the authors administered dexamethasone (a synthetic glucocorticoid) to pigs and examined changes in hepatic metabolism and gene expression mediated by the glucocorticoid receptor, a NR that regulates various hepatic genes upon binding to glucocorticoids. Dexamethasone administration led to changes in serum glucose and triglycerides as well as to altered expression of thousands of genes related to metabolism, immune response, cell growth, etc. Because treatment was given at two different doses, the authors were able to characterize a dose-dependent transcriptional response to dexamethasone. The authors show that immune-related genes respond to low doses of dexamethasone while carbohydrate metabolism-related genes only respond to higher doses.

The liver not only responds to hormonal signals but, as highlighted by Charni-Natan et al., also plays a central role in the metabolism of steroid hormones: the liver is responsible for: (a) Cholesterol synthesis, which is the major precursor for steroid hormones. (b) Production and secretion of steroid hormone carrier proteins. (c) Metabolizing steroid hormones by cytochrome P450 enzymes (CYP). (d) Production of several steroid-conjugating enzymes that facilitate steroid urinary excretion. Interestingly, the authors have summarized several studies showing a role for the p53 transcription factor in regulating hepatic genes responsible for steroid metabolism. The authors describe studies showing that p53 regulates CYP19 (responsible for transforming androgens to estrogens) as well as SHBG and CBG (two carrier proteins that facilitate transport of steroid hormone in circulation). Additional data are presented to suggest a role for p53 in regulating hepatic glucocorticoid metabolism. These data are especially intriguing considering that p53 is classically regarded as a tumor suppressor protein and a role for it regulating hepatic steroid metabolism is a nascent concept.

The review by Xia et al. focuses on estrogen-related receptor alpha (ERRα), a NR that plays a central role in energy metabolism. The ERRα ligand is unknown and several findings suggest that the principal regulation of ERRα activity is mediated by co-regulators, micro-RNAs, and post-translational modifications. Thus, ERRα can respond to upstream nutritional and hormonal stimuli not by direct activation, but via the crosstalk with its co-regulators that are themselves modulated by extracellular cues. The authors summarize ample data showing the role of ERRα in positively regulating oxidative gene expression, aerobic respiration and ATP synthesis. Accordingly, ERRα regulates many mitochondrial metabolism genes and is considered a “master regulator of the nuclear-encoded mitochondrial transcriptome.” The authors provide a useful resource listing ERRα target genes based on ERRα binding in the vicinity of the gene's promoters as well as the effect of ERRα manipulation on their expression levels.

Assembly of TFs at gene regulatory elements (enhancers) functions as a platform for recruitment of transcriptional co-regulator complexes facilitating post-translational modifications to histones, TFs and co-regulators, which in turn tunes transcription of associated genes. For example, acetylation of a number of histone lysine residues, catalyzed by histone acetyl transferases (HATs) and histone deacetylases (HDACs), is strongly associated with the level of nearby gene expression (4). Importantly, HATs and HDACs are part of larger protein complexes and structural members of these complexes are also implicated in hepatic metabolism as highlighted by Liang et al. This review provides comprehensive insights to the function of the NCoR complex in normal and diseased liver, including a summary of genetic studies attempting to understand the individual components of the NCoR complex. Importantly, these studies show a clear functional overlap between individual NCoR subunits in hepatic lipid metabolism. Yet a number of striking differences are also discussed, suggesting that individual subunits such as NCOR1, NCOR2, and GPS2 survey different TFs bound to enhancers thereby providing specificity to the complex. For example, GPS2 selectively regulates expression of genes involved in cholesterol homeostasis (via LXR), lipid metabolism (via PPARα) and inhibition of inflammatory responses (via LRH1) manifested in protection against non-alcoholic liver disease (NAFLD) when GPS2 is disrupted in the liver. In contrast, disruption of HDAC3, NCOR1, or TBL1 leads to increased hepatic lipid accumulation.

A number of NRs control expression of genes involved in hepatic metabolic homeostasis and thus are attractive drug targets for NAFLD and non-alcoholic steatohepatitis (NASH). As NAFLD and associated diseases continue to increase worldwide, we need a better understanding of NAFLD development and the progression of NAFLD into NASH. A study by Seda et al. has used a transcriptomics approach to study differential gene expression in liver biopsies from patients with NAFLD and non-steatotic liver. The authors sample liver biopsies from a cohort of adult liver transplant recipients as a part of follow-up surveillance. Thus, providing insights to a unique cohort. The authors found a correlation between gene expression and NASH score, hepatocyte ballooning, and inflammation. Moreover, pathway analysis of genes differentially regulated in the steatotic liver suggested dysregulated bile acid metabolism, glucagon signaling, amino acid metabolism, and increased inflammation. Using a bioinformatic approach, FXR was found as a potential regulator of many of these dysregulated pathways, emphasizing the clinical relevance of targeting this NR for treatment of NAFLD and NASH.

Insights to transcriptional regulation of liver biology has been accelerated by the development of Next Generation Sequencing technologies providing a wealth of genome-wide vistas to gene expression as well as to TF and co-regulator occupancy of the genome. Future challenges include a deeper understanding of the cooperative action of TFs and co-regulators in orchestrating hepatic gene regulatory programs. Moreover, with the recent development of single cell sequencing technologies and advanced computational approaches we are also starting to gain insights to liver biology at cellular resolution (5–8) which likely will provide novel drug targets for treatment of diseases associated with a dysfunctional liver.

## Author Contributions

All authors listed have made a substantial, direct and intellectual contribution to the work, and approved it for publication.

### Conflict of Interest

The authors declare that the research was conducted in the absence of any commercial or financial relationships that could be construed as a potential conflict of interest.
